# pH-Sensitive and Long-Circulation Nanoparticles for Near-Infrared Fluorescence Imaging-Monitored and Chemo-Photothermal Synergistic Treatment Against Gastric Cancer

**DOI:** 10.3389/fphar.2020.610883

**Published:** 2020-11-26

**Authors:** Yun Zhou, Xuanzi Sun, Liansuo Zhou, Xiaozhi Zhang

**Affiliations:** ^1^ Department of Radiation Oncology, The First Affiliated Hospital of Xi’an Jiaotong University, Xi’an Jiaotong University, Xi’an, China; ^2^ College of Clinical Medicine, Xi’an Medical University, Xi’an, China

**Keywords:** gastric cancer, photothermal therapy, chemotherapy, synergistic treatment, continuous imaging

## Abstract

Gastrectomy is the primary therapeutic option for gastric cancer. Postoperative treatment also plays a crucial role. The strategy to improve the postoperative prognosis of gastric cancer requires a combined system that includes a more efficient synergistic treatment and real-time monitoring after surgery. In this study, photothermal-chemotherapy combined nanoparticles (PCC NPs) were prepared via π-π stacking to perform chemo-photothermal synergistic therapy and continuous imaging of gastric cancer. PCC NPs had a spherical morphology and good monodispersity under aqueous conditions. The hydrodynamic diameter of PCC NPs was 59.4 ± 3.6 nm. PCC NPs possessed strong encapsulation ability, and the maximum drug loading rate was approximately 37%. The NPs exhibited extraordinary stability and pH-response release profiles. The NPs were rapidly heated under irradiation. The maximum temperature was close to 58°C. PCC NPs showed good biocompatibility both *in vitro* and *in vivo*. Moreover, the NPs could effectively be used for *in vivo* continuous monitoring of gastric cancer. After one injection, the fluorescent signal remained in tumor tissues for nearly a week. The inhibitory effect of PCC NPs was evaluated in a gastric cancer cell line and xenograft mouse model. Both *in vitro* and *in vivo* evaluations demonstrated that PCC NPs could be used for chemo-photothermal synergistic therapy. The suppression effect of PCC NPs was significantly better than that of single chemotherapy or photothermal treatment. This study lays the foundation for the development of novel postoperative treatments for gastric cancer.

## Introduction

Gastric cancer has one of the highest incidences of malignant gastrointestinal tumors worldwide. Globally, the morbidity and mortality of gastric cancer rank fifth and third, respectively (Song et al., 2017; Sigel et al., 2020). Effectively early diagnosis of gastric cancer is difficult to achieve. Multiple factors are related to gastric cancer (Yang et al., 2017). The therapeutic strategy for gastric cancer mainly depends on surgical treatment, which includes local excision, partial gastrectomy, and total gastrectomy. Chemotherapy is primary supplementary treatment postoperatively. Currently, surgery combined with postoperative chemotherapy for gastric cancer patients improves prognosis in early stage gastric cancer; however, the prognosis of intermediate and advanced gastric cancer remains unsatisfactory (Kang et al., 2015; Zuo et al., 2017). The strategy of further improving the comprehensive efficacy of gastric cancer involves a synergistic system that includes more efficient early diagnosis, monitoring, and synergistic treatment after surgery. Local thermotherapy is an adjuvant method to treat cancer. The majority of cancers are heat sensitive, and apoptosis is triggered at temperatures over 43°C (Hildeberandt et al., 2002). Photothermal therapy (PTT) is a novel noninvasive therapeutic technology. Photothermal agents are heated under irradiation and cause ablation of tumor tissues (Shibu et al., 2013). In addition, PTT can be used in combination with other treatments, such as chemotherapy, photodynamic therapy, immunotherapy, and radiotherapy (Sherlock et al., 2011; Sahu et al., 2013; Wang et al., 2013; Guo et al., 2014; Yi et al., 2015). Photothermal therapy combined with chemotherapy is an effective method to achieve synergistic therapy of gastric cancer because heat can promote release and endocytosis and reduce resistance (Shi et al., 2012). Moreover, dynamic monitoring postoperatively is also an important part of the synergistic system and can be used to assess tumor to guide the appropriate treatment schedule (Dhar et al., 2000; Zhang et al., 2004).

The functional agent is the core of the thermal therapy, and various materials are used for thermal therapy (Huang et al., 2006; Abadeer and Murphy, 2016; Liu et al., 2016). However, in recent decades near-infrared (NIR) dyes have been of interest in PTT against cancer, because they have strong photothermal effects, low toxicity, and appropriate excitation wavelengths (Zheng et al., 2011). Furthermore, the fluorescence of the NIR fluorescent dye also allows excellent real-time monitoring of tumor progress. At present, many NIR fluorescent dyes are used in clinical medicine and achieve a certain effect. Among them, indocyanine green (ICG) has been approved by the FDA for clinical application. However, defects ultimately limit the further utilization of ICG, including its poor solubility, low bioavailability, and concentration-related aggregation (Bahmani et al., 2013). Anthocyanin dye has been used as a fluorescent probe for *in vivo* tumor imaging (Wang et al., 2016; Li et al., 2017). IR-820 is a novel anthocyanin with properties similar to those of IGC but exhibits better stability and photothermal effects; thus, IR-820 can be used for tumor photothermal therapy. In addition, IR-820 can emit NIR fluorescence under irradiation, indicating that the dye can be used for tumor monitoring (Li et al., 2016; Zhang et al., 2018; Dong et al., 2019). However, IR-820 is also problematic in clinical applications. When IR-820 is combined with other medicines, such as chemotherapeutic agents, it may have an efficient synergistic antitumor effect. Doxorubicin (DOX) is commonly used in clinical cancer chemotherapy and also in combination with other drugs or agents for synergistic treatment (Bao et al., 2016; Zhao et al., 2016). Mechanistically DOX and IR-820 have great potential and complementarity in the treatment of gastric cancer. First, DOX has severe systemic toxicity and susceptibility to drug resistance (Xiong et al., 2010). IR-820 can be heated under irradiation; the hyperthermy can promote the accumulation of the drug and reduce the occurrence of resistance. Thus, the effective dosage of DOX can be significantly decreased in treatment and its side effects can be reduced. However, DOX and IR-820 possess distinct pharmacokinetic properties that are difficult to achieve simultaneously with good accumulation and performance in tumor tissues and will be an insurmountable limitation in clinical applications. Therefore, *in vivo* codelivery and long-term stability of the two agents are crucial technical issues to be solved in synergistic treatment of gastric cancer.

Developing and applying nanotechnology bring great benefit to medicine. However, the drug loading of common nanocarriers is often less than 10%. This disadvantage limits the further development of nanocarriers (Choi et al., 2011; Chen et al., 2015). Some physical bonding effects have been attempted to strengthen the effect on the construction of nanocarriers (Ke et al., 2014; Zhang et al., 2017). π-π stacking, which is similar to hydrogen bonding, is a noncovalent interaction between aromatic nuclei. Recently, π-π stacking technology has become an attractive method for molecular assembly, especially in the self-assembly of nanoparticles and drug delivery systems. This method does not destroy the properties of drugs and can improve their bioavailability (Shi et al., 2015). The majority of antitumor drugs are known to contain complex aromatic structures, which lead to poor water solubility and low bioavailability. Hence, π-π stacking can be used to assemble drug-loaded complexes for these drugs (Wei et al., 2016; Wang et al., 2017; Zhuang et al., 2019). Hennink and colleagues prepared a polymeric micelle via π-π stacking for paclitaxel delivery; the drug loading rate reached 23%, which is well above that of the clinical paclitaxel medicine Genexol-PM (Shi et al., 2013). He et al. developed a series of micelles via π-π stacking and found that their ability for DOX loading was proportional to the aromatic ring (Liang et al., 2015). Zhang et al. prepared multidrug loading nanocarriers via π-π stacking for combination chemotherapy (Zhang et al., 2015). Both DOX and IR-820 have aromatic nuclei in their molecular structures, which means that these agents can assemble via π-π stacking. For the assembly material, we considered dopamine. Dopamine is a small compound with a benzene ring and possesses excellent biocompatibility and stability. Dopamine can combine with DOX and IR-820 in an alkaline aqueous solution through π-π stacking and form stable drug-loaded NPs. The prepared NPs not only extend the circulation time of the payloads, but also perform tumor targeted delivery through enhanced permeability and retention (EPR) effects (Peer et al., 2007; Jain and Stylianopoulos, 2010).

In the present study, a multifunctional nanocarrier was constructed for chemo-photothermal synergistic therapy and dynamic monitoring in gastric cancer. The materials that were used are dopamine and poloxamer F127, which is a surfactant for hydrotropy. The agents were DOX and IR-820, which have chemotherapeutic effects and can be used for simultaneous photothermal treatment and *in vivo* fluorescent imaging. The morphology, encapsulation, and stability were initially of the PCC NPs evaluated. Subsequently, the toxicity, targeted delivery, and tumor suppression of the PCC NPs were determined in cell lines and a BALB/c mouse model. The results could provide a theoretical foundation for the postoperative treatment of gastric cancer.

## Materials and Methods

### Materials

Dopamine hydrochloride (98%), poloxamer F-127, DOX, and tris(hydroxymethyl)aminomethane (Tris) were purchased from Sigma-Aldrich Corp. (MO, USA). IR-820 was purchased from Aladdin Crop. (Shanghai, China). The antibiotics, trypsin, CCK-8 kit, Annexin V-FITC apoptosis kit, and DAPI kit were purchased from Beyotime Co., Ltd. (Shanghai, China). HL-7702 (human normal liver cells), IMR-90 (human embryonic lung fibroblast cells), and HUVECs (human umbilical vein endothelial cells) were purchased from Tongpai Co., Ltd. (Shanghai, China). Human gastric cancer cell lines (MKN45, BGC-823 and SGC-7901) were purchased from Huiying Co., Ltd. (Shanghai, China). DMEM high glucose medium and fetal bovine serum (FBS) were purchased from Thermo Fisher Inc. (MA, USA). Other reagents were supplied by Sinopharm Crop. (Beijing, China). BALB/c mice and BALB/c-nu/nu mice were purchased from Tengxin Co., Ltd. (Chongqing, China).

### Preparation of PCC NPs

Construction of PCC NPs is a self-assembly process that depends on π-π stacking of dopamine, DOX, and IR-820 under alkaline conditions. The methods were reported by Wang et al. (2018). Initially, 18 ml of Tris solution, pH 8.8, was poured into a flask. Subsequently, 18 mg of dopamine and 1 ml of a poloxamer and IR-820 mixed solution (DMSO; poloxamer, 10 mg/ml; IR-820, 20 mg/ml) were dropped into the flask under stirring. The 100 μL of DOX solution (20 mg/ml) was added to the mixture solution, and stirring was maintained for 3 min. The mixture was ultrasonically treated for 10 min. Then, mixture was incubated for 72 h at 28°C in a rotary stirrer, and PCC NPs were obtained. The NPs were further purified by dialysis and centrifugation to remove unreacted materials. The PCC NPs dispersed liquid was concentrated to 10 mg/ml by centrifugation.

### Characterization of PCC NPs

The hydrodynamic diameter of PCC NPs was initially measured by dynamic light scattering using a Malvern instrument (Malvern, Ltd., UK). The morphology and size of PCC NPs were observed by TEM (JEOL Corp., Japan). The encapsulation ability of PCC NPs was further evaluated. The total mass of PCC NPs was measured by residue of freeze-drying method. The DOC or IR-820 in residue was redissolved by DMSO and the concentrations were measured via fluorescence spectrometry. The encapsulation rate (ER) and drug loading rate (DL) of DOX and IR-820 were calculated by total mass of PCC NPs and input and remaining mass of agents. The *in vitro* stability of PCC NPs was evaluated according to the changing of hydrodynamic size under various conditions, including PBS, complete medium, and FBS at 4°C or 37°C. *In vitro* drug release was evaluated by dialysis. The PCC NPs dispersion liquid was enclosed in dialysis bags, with a molecular weight cutoff of 2000 Da. Then the dialysis bags were assigned in various experiments. First, the release of PCC NPs was tested in PBS at 4°C and free DOX at the same concentration as the control. The pH-response of PCC NPs was measured in acetate buffered saline at pH 7.4, pH 6.5, and pH 5.2. The effect of temperature on the release of DOX was evaluated in PBS at 4, 37, 45, and 58°C. The release rates were determined and calculated by concentrations of DOX in the outer phase of dialysis.

### Photothermal Conversion of PCC NPs

The PCC NPs dispersion liquid (500 μl, 50 μg/ml) was added to a 1.5 ml tube. Then the tube was continuously irradiated with 808 nm laser at a power density of 1 W/cm^2^, and the temperature change was measured using an infrared thermometer at 0, 1, 2, and 3 min. The photothermal conversion in different concentrations of PCC NPs was also evaluated in a 1.5 ml tube with the same laser irradiation parameters. The concentrations of PCC NPs were 10, 20, 30, and 40 μg/ml. To evaluate the photothermal effect of PCC NPs under different conditions, the NPs were dispersed in PBS, complete medium, and FBS, and irradiation was performed at a power density of 1 W/cm^2^ for 3 min. The temperature changes were also determined using an infrared thermometer. The photothermal effect was further evaluated in mouse. Fifty microliters of the PCC NPs dispersion liquid at a concentration of 40 μg/ml was subcutaneously injected into the right crotch of a BALB/c-nu/nu mouse. Then the injection area was irradiated by the laser at a power density of 1 W/cm^2^, and the temperature change was determined at different time points. Moreover, the photothermal conversions of the materials were also measured. The photothermal curve of PCC NPs was plotted according to the temperature values before and after switching off the laser.

### Cell Internalization of PCC NPs

DOX emits red fluorescence, which could be used to directly observe internalization and intracellular release of PCC NPs. SGC-7901 cells were seeded into 3.5 cm confocal dishes at a density of 1.5×10^5^ cells/dish and incubated at 37°C under 5% CO_2_ for 24 h. When cell adherence and growth were good, PCC NPs were added to dishes. Subsequently, the cells were treated with a 4% paraformaldehyde solution for different times, and cell nuclei were stained with a DAPI kit. All samples were observed with a confocal microscope (TCS SP5 II, Leica, Germany). The fluorescent intensities in cells were measured using ImageJ software.

### Cytotoxicity of PCC NPs

A CCK-8 assay was employed to evaluate the cytotoxicity of PCC NPs in various cells, including normal human cells (HUVECs, IMR-90, and HL-7702) and human gastric cancer cell lines (BGC-823, SGC-7901, and MKN45). The logarithmic phase cells were seeded into 96-well plates at a density of 8 × 10^3^ cells/well. Subsequently, PCC NPs, IR-820, and DOX at different concentrations were added to the wells. The plates were incubated at 37°C under 5% CO_2_. The cell densities were continuously observed via microscopy (Nikon, Japan). After 24 h of coincubation, the wells were replaced with a fresh colorless medium containing 10% CCK-8 solution. The incubation was extended for 2 h, and then the wells were measured at 450 nm absorbance with a microplate reader (Varioskan LUX, ThermoFisher, USA). The cell viabilities were calculated with GraphPad Prism 5.0 software.

### *In vitro* Antitumor Effect of PCC NPs

SGC-7901 cells were seeded into 3.5 cm dishes at a density of 2×10^5^ cells/dish. When the cells in the dish grew to over 90% confluence, PCC NPs were added. The dish was then incubated at 37°C under 5% CO_2_ overnight. Then stale medium was replaced by fresh colorless medium, and aluminum foil was used to cover half of the dish. Then the dish was exposed to 808 nm laser irradiation at a power density of 1 W/cm^2^ for 8 min. The cellular morphology on both sides of the cover line was observed with a microscope (Nikon, Japan). Meanwhile, a CCK-8 assay was also used to evaluate the effects of the photothermal treatment in SGC-7901, BGC-823, and MKN45 cells. Logarithmic phase cells were seeded into 96-well plates at a density of 8 × 10^3^ cells/well. Subsequently, PCC NPs at different concentrations were added to the wells and incubated at 37°C under 5% CO_2_ for 24 h. The wells were replaced with fresh colorless medium, and an 808 nm laser was used at a power density of 1 W/cm^2^ for 5 min in each well. After incubation for 1 h, the cell viabilities were measured and calculated with the method described above.

A colony formation assay was used to evaluate cell proliferation under photothermal treatment. SGC-7901, BGC-823, or MKN45 cells were seeded into 6 cm dishes at a density of 500 cells/dish. Then PBS, IR-820, DOX, and PCC NPs were added to dishes. The dishes were incubated at 37°C under 5% CO_2_ for 48 h. In the photothermally treated group, PCC NPs or IR-820 were added to the cells and incubated for 8 h. Then the cells were suspended at a density of 500 cells/ml. One milliliter of the cell suspension was added to a 1.5 ml tube and 808 nm laser irradiation was performed at a power density of 1 W/cm^2^ for 5 min. Then, the cells were seeded into a 6 cm dish and incubated for 48 h. Subsequently, stale medium was replaced with fresh medium containing 20% FBS. Cells were incubated for another 5 d and treated with fix-stain buffer (5% Coomassie brilliant blue in methanol) for 15 min. The stained cell colonies were counted.

Flow cytometry was used to determine the apoptosis of SGC-7901 cells under treatments. The cells were seeded into 24-well plates at a density of 1 × 10^5^ cells/well. PCC NPs were added to the dish and incubated for 8 h. Then the dish was treated with 808 nm laser irradiation for 3 min and further incubated for 4 h. The cells were collected and stained with an Annexin V-FITC apoptosis kit. In the control groups, the dishes were treated with DOX or IR-820. The cells were analyzed using flow cytometry (Accuri C6, BD, USA).

### Hemolysis Assay

PCC NPs were administered via intravenous injection. Therefore, toxicity was initially evaluated via a hemolysis assay. Red blood cells of mice were collected for the experiment. The cells were dispersed in PBS at a density of 2% and infused into 1.5 ml tubes. Subsequently, Triton X-100 (10 mg/ml), PCC NPs (30 mg/ml), DOX (1 mg/ml), and IR-820 (10 mg/ml) were added to the tubes. All tubes were placed in a 37°C water bath for 2 h. Then, the cell suspensions were centrifuged. The supernatants were used to measure absorbance at 394 nm and the lysis ratio was calculated.

### *In vivo* Acute Toxicity of PCC NPs

Fifteen female and fifteen male BALB/c mice were utilized to evaluate the acute toxicity of PCC NPs *in vivo*. The mice were fed in a SPF animal room for 1 week to acclimate. Then, the mice were randomly divided into three groups of five females and five males in each group. The PCC NPs, IR-820, and DOX were intravenously injected into respective groups. The dose of PCC NPs was 100 mg/kg, DOX was 3 mg/kg, and IR-820 was 30 mg/kg. The survival rate was recorded during 2 weeks. After euthanasia, the hearts, livers, and kidneys of mice were collected for pathological analysis. All animal experiments in this study were approved by the Laboratory Animal Administration Committee of Xi’an Medical University. The protocols for animal experiments followed the Guidelines for the Use and Care of Experimental Animals at Xi’an Medical University. The Animal Ethics Approved Document Number is XY-AUC-2019-168.

### Xenograft Mouse Model

Male BALB/c-nu/nu mice were used for the preparation of the gastric cancer xenograft model. The mice were fed in a SPF animal room for 1 week to acclimate. SGC-7901 cells were digested with 0.25% trypsin and cell suspension was prepared at a density of 5 × 10^6^ cells/ml. One hundred microliters of the cell suspension was subcutaneously injected into the crotch of the mouse. When the tumor grew to an appropriate size, the mice were used as a xenograft model for *in vivo* experiments.

### *In Vivo* Distribution of PCC NPs

IR-820 is a NIR fluorescent dye that can be used to monitor PCC NPs. Two xenograft models were chosen to determine the *in vivo* distribution of PCC NPs. Two hundred microliters of the PCC NPs dispersed liquid with 20 μg/ml IR-820 was intravenously injected into mice. Another mouse was injected with an equal concentration of IR-820 solution as the control. Fluorescent signals were observed with an IVIS instrument (Perkin Elmer, MA, USA) at sequential time points after injection. Mice were euthanized after observation, and their organs and tumors were collected. The tissues were observed using an IVIS instrument with the same parameters.

### *In Vivo* Antitumor Evaluation of PCC NPs

Twenty xenograft models with average tumor volume of approximately 100 mm^3^ were utilized to evaluate the *in vivo* antitumor effect of PCC NPs. The mice were randomly divided into four groups. These groups were treated with 1) saline; 2) DOX; 3) IR-820 + laser; and 4) PCC NPs + laser. The dose of DOX was 0.5 mg/kg, IR-820 was 5 mg/kg, and dose of PCC NPs was 15 mg/kg. Laser irradiation was performed at a power density of 1 W/cm^2^ for 3 min and the irradiating distance was 5 cm. The irradiation intensity was verified to not cause obvious burns on the mouse skin in experiment. The route of administration of the PCC NPs was intravenous injection. The interval time of laser treatment was based on the results of the *in vivo* distribution. The tumors were photographed and their size was measured every 5 days. After 30 days, the mice were euthanized by CO_2_ overdose. Tumor tissues were collected and weighed.

### Statistical Analysis

Two-way ANOVA and Student’s *t*-test were used in statistical analyses performed using GraphPad Prism 5.0 software. The data are presented as the mean values ± standard deviation (SD) of independently repeated experiments. A *p* value <5 indicated that the data were significantly different.

## Results

### Preparation and Characterization of PCC NPs

**FIGURE 1:**
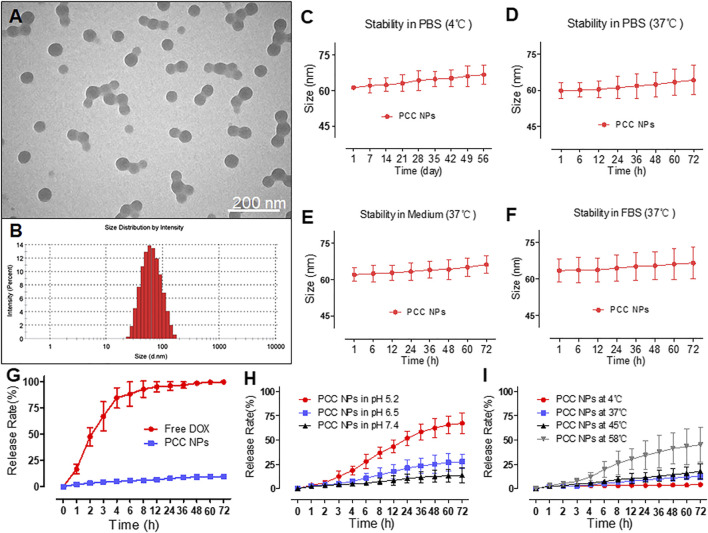
Characterization of PCC NPs. Morphology of PCC NPs under TEM observation **(A)**; hydrodynamic size of PCC NPs **(B)**; *in vitro* stability of PCC NPs in PBS at 4°C **(C)**; *in vitro* stability of PCC NPs in PBS at 37°C **(D)**; *in vitro* stability of PCC NPs in completed medium at 37°C **(E)**; *in vitro* stability of PCC NPs in FBS at 37°C **(F)**; *in vitro* drug release of PCC NPs **(G)**; *in vitro* pH-response drug release of PCC NPs **(H)**; *in vitro* temperature-related drug release of PCC NPs **(I)**. Error bars represent the SD of the mean.

PCC NPs exhibited a spherical morphology and good monodispersity in TEM observation. The average size of the NPs was approximately 60 nm (Figure 1A). The hydrodynamic diameter was 59.4 ± 3.6 nm, which was mutually confirmed by TEM observation. Figures 1C–F show the stability of PCC NPs under different conditions. The colloidal stability was evaluated by the change of hydrodynamic diameter. Within 8 weeks, the size of the PCC NPs did not exhibit an obvious change in PBS at 4°C. The results indicated that PCC NPs could be effectively stored in PBS. Subsequently, PCC NPs were evaluated in PBS, complete medium, and FBS at 37°C. The aim was to determine whether the NPs could be used in further *in vitro* and *in vivo* experiments. The hydrodynamic size was extremely stable in all the solution environments. Although the average diameters were not significantly changed, an increase in the size distribution interval was the only matter worthy of attention. This phenomenon indicated that PCC NPs efficiently disperse under physiological conditions and could be utilized in subsequent experiments. The encapsulation ability of PCC NPs was evaluated via ER and DL. The maximum ER and DL of DOX were 96.7 ± 2.4 and 3.6 ± 0.9%, respectively. Meanwhile, the maximum ER and DL of DOX were 97.2 ± 1.8 and 34.1 ± 2.5%, respectively. The mass ratio of DOX and IR-820 in PCC NPs was approximately 1:10. The proportion was consistent with the input of DOX and IR-820, which further demonstrated that the NPs possess a superior encapsulation ability.

### *In Vitro* Release Profile of PCC NPs

To verify control and sustained release of PCC NPs, we determined the release profile. Figure 1G shows the normal release process in PBS at room temperature. Free DOX was used as the control and exhibited obvious burst release characteristics, with the majority of DOX released within 8 h. By comparison, DOX in PCC NPs showed a very sustained release profile. Less than 10% of the payloads were released into the outer phase of dialysis. Then, the pH-response release of PCC NPs was evaluated. The results are shown in Figure 1H. PCC NPs exhibited a significant distinction of release under different pH conditions. As mentioned above, π-π stacking can only occur under alkaline conditions. When the pH was 7.4, the release of PCC NPs was obviously slower than at pH 6.5 and pH 5.2. These two pH values match the tumor microenvironment and interior of the lysosome. A pH 7.4 is a common physiological condition *in vivo*. The results indicated that PCC NPs undergo a pH-response release. As photothermal NPs, PCC NPs will appear at high temperatures. As shown in Figure 1I, the release of PCC NPs at 58°C was much faster than that at 4, 37, and 45°C. Moreover, even at 58°C, the release rate was still lower than 50%. Among the temperatures, 4°C is commonly used in storage, and 37°C is the normal physiological temperature *in vivo*. The temperature of 45°C is usually used for tumor thermotherapy, while 58°C is the maximum photothermal conversion temperature of PCC NPs. The results indicated PCC NPs can be stably released at different temperatures.

### Photothermal Conversion of PCC NPs

**FIGURE 2:**
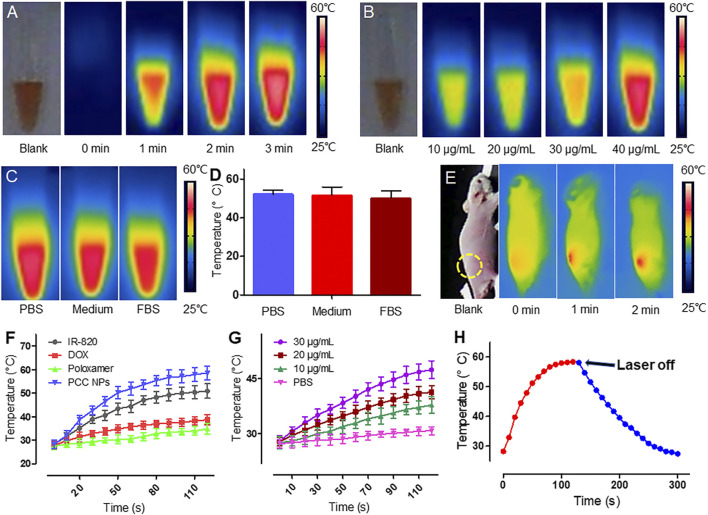
Photothermal conversion of PCC NPs. Infrared thermal imaging of PCC NPs under irradiation at different times **(A)**; infrared thermal imaging of PCC NPs under irradiation at different concentrations **(B)**; photothermal effect of PCC NPs in different solution with irradiation time of 3 min **(C** and **D)**; infrared thermal imaging of mouse that was subcutaneously injected PCC NPs under irradiation **(E)**; photothermal performance of PCC NPs and compositions **(F)**; temperature changing curves of PCC NPs at different concentrations **(G)**; temperature change of PCC NPs before and after switching off the irradiation **(H)**. All of the samples were irradiated with 808 nm laser at a power density of 1 W/cm^2^. Error bars represent the SD of the mean.

Photothermal conversion is another core performance of PCC NPs and is combined with chemotherapy for the synergistic treatment of gastric cancer. Figure 2 shows the *in vitro* photothermal effect of PCC NPs. Figure 2A shows the temperature increase of 50 μg/ml PCC NPs under 808 nm laser irradiation. PCC NPs exhibited a rapid heating effect under irradiation. The maximum temperature reached 58°C over 3 min. The concentration-related photothermal effect is shown in Figure 2B. Photothermal effect of PCC NPs exhibited an obvious correlation with concentration. Nevertheless, the increase was not obvious at low concentrations. This phenomenon was further verified in the temperature curve (Figure 2G). The increase in temperature only reaches 45°C under 30 μg/ml. The primary reason of this result is that the maximum temperature of PCC NPs was only approximately 58°C. However, the temperature increase is very appropriate, as it fits the requirements for *in vivo* tumor thermotherapy well. PCC NPs were verified to be effectively dispersed in various solutions. The effects of photothermal conversion in the different aqueous conditions were evaluated. The results are shown in Figures 2C,D. There was no significant difference between PCC NPs dispersed in PBS, medium, and FBS. All groups could approximately reach the maximum temperature. Subsequently, the temperature increase of transdermal irradiation was determined in a BALB/c-nu/nu mouse. As shown in Figure 2E, the temperature in the PCC NPs injection area was obviously increased under irradiation at a power density of 1 W/cm^2^ for 2 min. The maximum temperature reached approximately 47°C, which is well suited for *in vivo* tumor thermotherapy. Figure 2F shows the difference between PCC NPs and other materials. DOX and poloxamer do not have photothermal effects; thus, there were no obvious temperature increases in either group. The temperature slightly increased in the DOX solution under laser irradiation. The increase in temperature occurred more significantly in PCC NPs than in the IR-820 group. The primary reason for this difference is that polydopamine (PDA) also has a certain effect on photothermal conversion. Figure 2H indicates that the temperature increase was caused by irradiation. When the laser irradiation was switched off, the temperature rapidly decreased. The results amply demonstrate that PCC NPs possess excellent photothermal effects, which are appropriate in the treatment of gastric cancer.

### *In vitro* Internalization of PCC NPs

**FIGURE 3:**
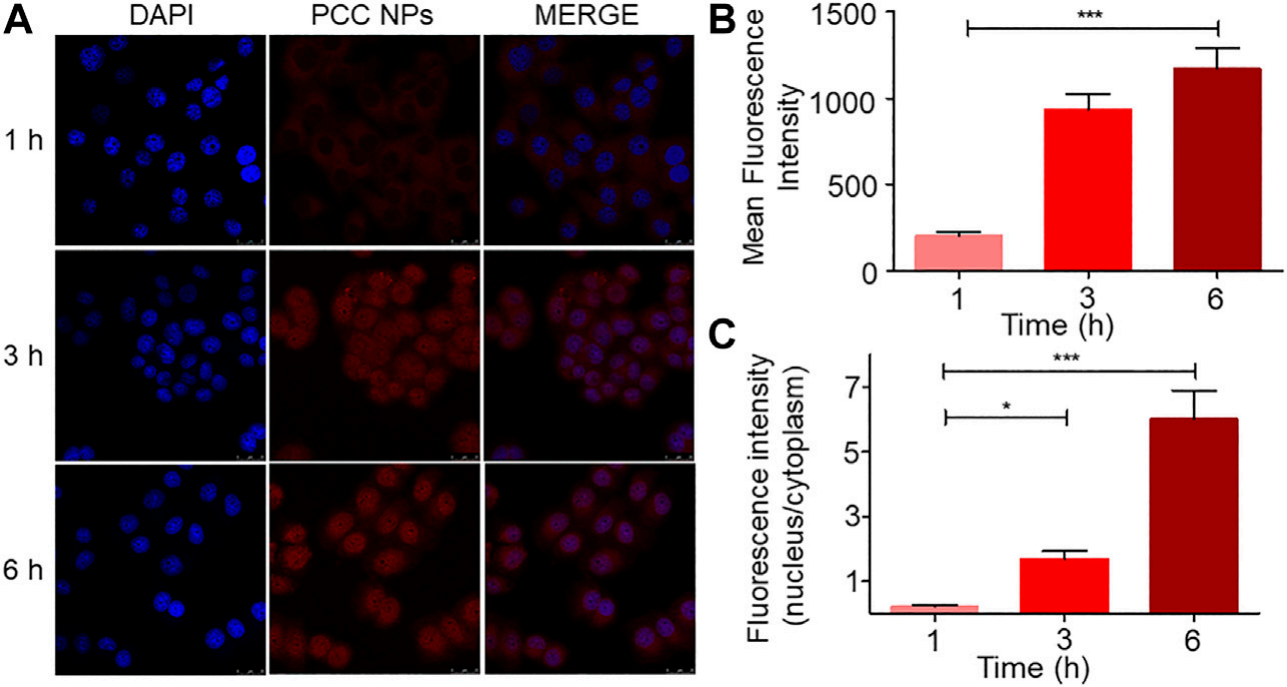
Internalization and intracellular release profile of PCC NPs in SGC-7901 cells. The distribution of fluorescence in cells **(A)**; average fluorescence intensity in cell **(B)**; ratio of the fluorescence intensity between nuclei and cytoplasm **(C)**. Error bars represent the SD of the mean. * indicates *p* < 0.05, *** indicates *p* < 0.001.

The cell internalization profile is shown in Figure 3. The red and blue fluorescence originated from DOX and DAPI-stained cell nuclei, respectively. As shown in Figure 3A, the fluorescent signal of DOX was gradually enhanced in cells, especially in nuclei. Initially, after 1 h, there was scarce red fluorescence accumulated in the cell, concentrated mainly in the cytoplasm. After 3 h, the red fluorescent signal was spread all throughout the cell, and more remarkably, some nuclei exhibited higher accumulation than the cytoplasm. At 6 h of treatment, the red fluorescent signal was more obvious and mainly accumulated in cell nuclei. Quantitative analysis of the fluorescent signal further verified this phenomenon. The mean fluorescence intensity gradually increased in the cell (Figure 3B). However, a different increase of the signal occurred between the nuclei and cytoplasm. As Figure 3C shows, the fluorescence ratio between nuclei and the cytoplasm exhibited a different variation trend compared with the fluorescent signal. The ratio indicated that more DOX accumulated in cell nuclei over time. This phenomenon suggested that PCC NPs could steadily release the payloads in the cell.

### *In Vitro* Cytotoxicity and Suppression Effect of PCC NPs

**FIGURE 4:**
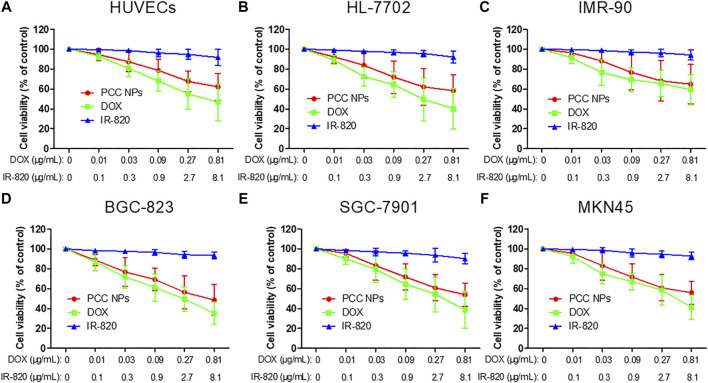
*In vitro* cytotoxicity of PCC NPs. Human umbilical vein endothelial cell, HUVECs **(A)**; human normal liver cell, HL-7702 **(B)**; human embryonic lung fibroblasts cells, IMR-90 **(C)**; human gastric cancer cell, BGC-823, SGC-7901, and MKN45 **(D**–**F**). Error bars represent the SD of the mean.

To evaluate the cytotoxicity of PCC NPs without laser irradiation, a CCK-8 assay was employed in the experiment. Three normal human cell lines: HUVECs, IMR-90, and HL-7702, were initially incubated for determination of cytotoxicity. These cell lines were derived from the vascular endothelium, lung, and liver and were used to evaluate potential toxicity in normal organs and tissues. Meanwhile, three gastric cancer cell lines (BGC-823, SGC-7901, and MKN45) were also utilized in the measurements. The results are shown in Figure 4. The concentration ratio of DOX and IR-820 was 1:10, meeting the proportion in PCC NPs. All of the cells were insensitive to IR-820 treatment. This result indicated that IR-820 has low cytotoxicity. DOX treatments exhibited more cytotoxicity than the PCC NPs treatment groups. However, there was no significant difference between the two groups. The results suggested that PCC NPs could perform chemotherapeutic effect of DOX. The possible reason for the lower cytotoxicity of PCC NPs than DOX is that DOX in PCC NPs is more slowly released in cells. Their biocompatibility was initially verified by these *in vitro* experiments.

**FIGURE 5:**
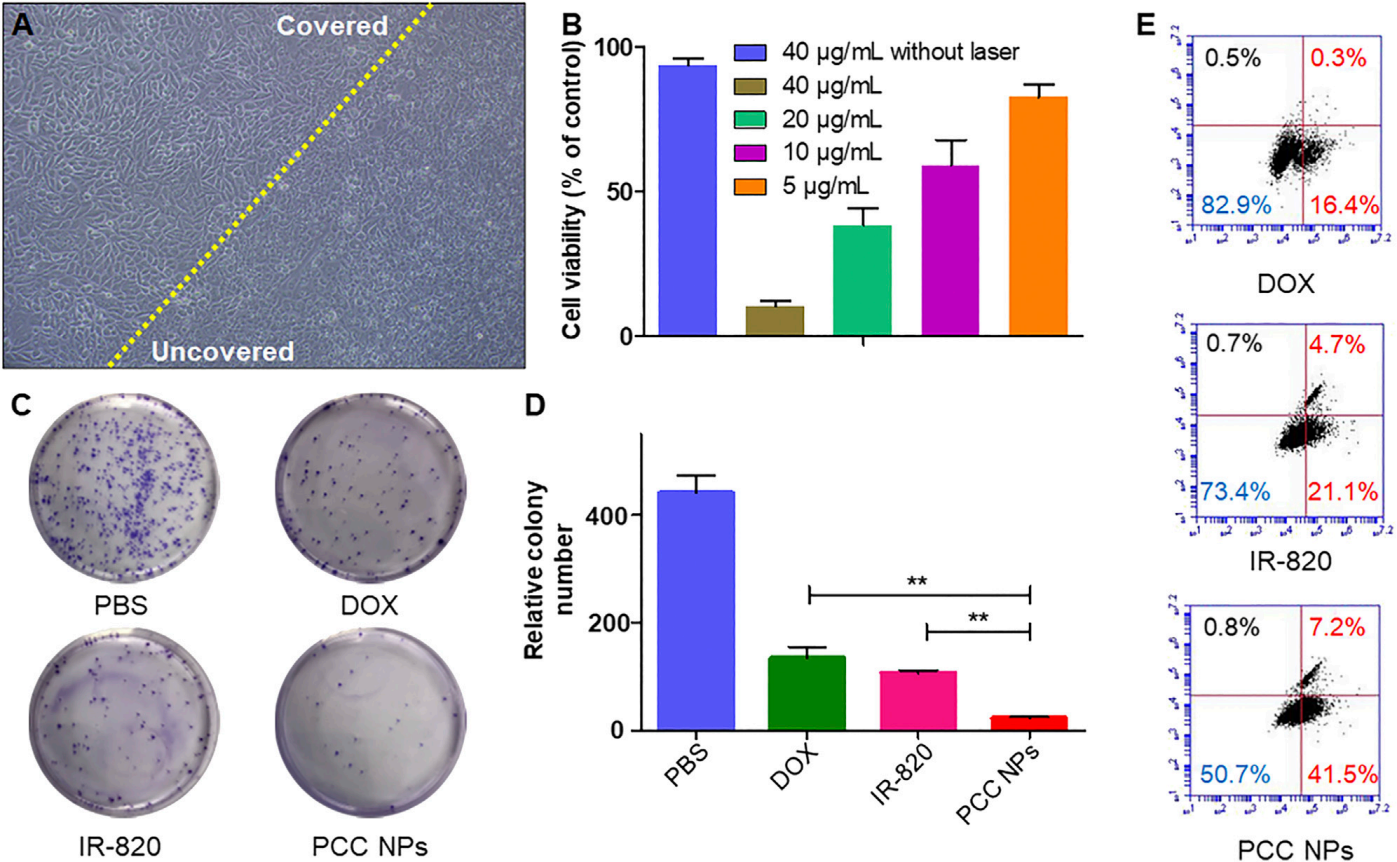
*In vitro* antitumor effect of PCC NPs. Directly damage cells under photothermal treatment, the cells on the left of the yellow line were untreated, and the right side was treated with PCC NPs under irradiation **(A)**; the cell viability at different concentrations of PCC NPs under photothermal treatment **(B)**; the results of colony formation assays. Cells in the PCC NPs and IR-820 treated groups were irradiated after the administration of samples **(C, D)**; flow cytometry assay of cells treated with PCC NPs, IR-820, or DOX **(E)**. Error bars represent the SD of the mean. ** indicated *p* < 0.01.

**FIGURE 6:**
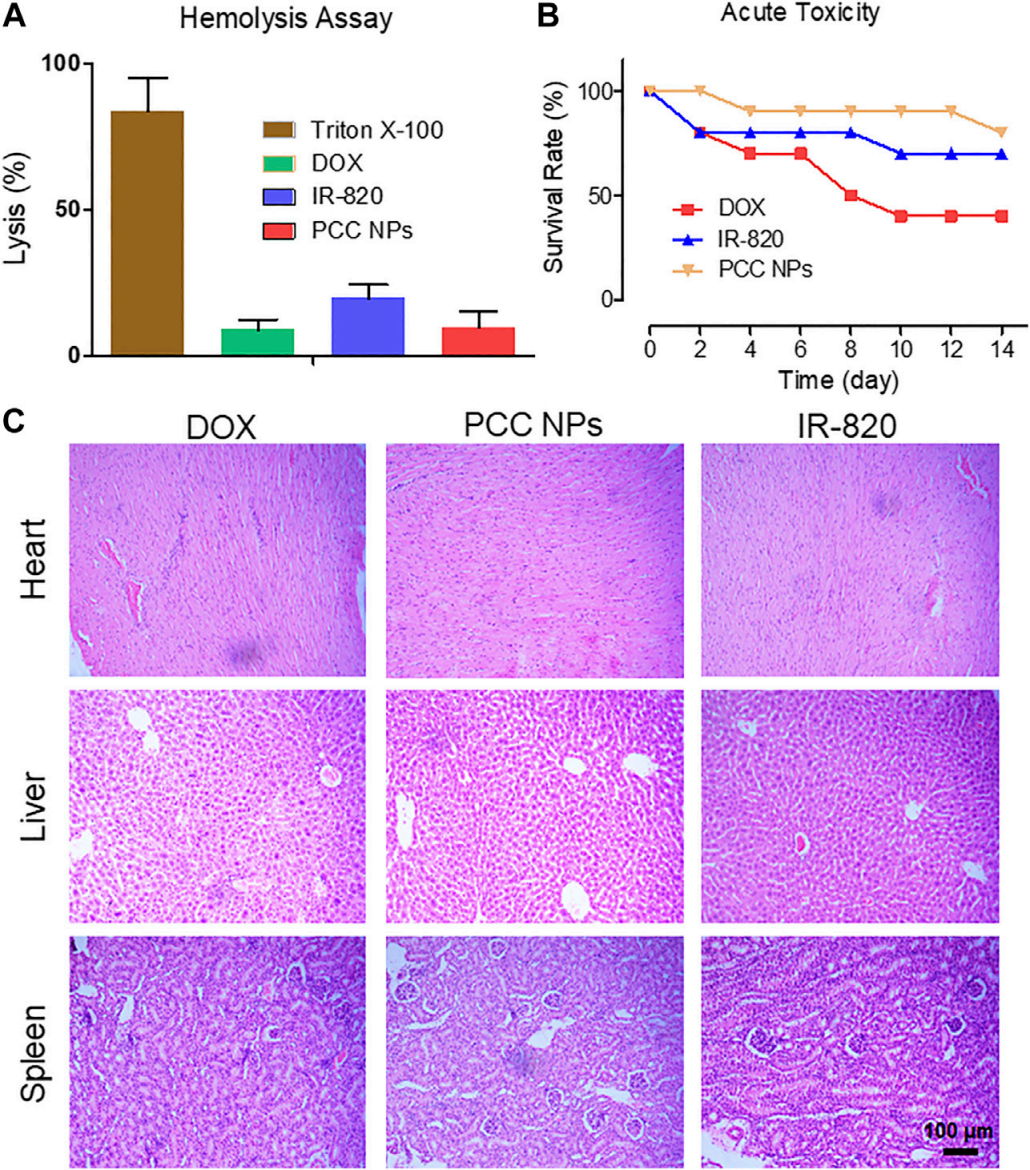
*In vivo* toxicity evaluation of PCC NPs. The hemolysis ratio in the PCC NPs, IR-820, and DOX treated groups **(A)**; survival rate of mice in the different treatment groups **(B)**; pathological characteristics of the heart, liver, and kidney in the different treatment groups **(C)**. Error bars represent the SD of the mean.

*In vitro* photothermal treatment was evaluated using the gastric cancer cell lines SGC-7901, BGC-823, and MKN45. Figure 5A shows direct SGC-7901 cell damage with PCC NPs under laser irradiation. The morphology of cells in the untreated area showed obvious abnormalities. By comparison, a significant morphological change did not occur in cells in the covered area. Subsequently, the treatment effects under different concentrations of PCC NPs were evaluated by the CCK-8 assay. The results are shown in Figure 5B and Supplementary Figures S1A, C. There was no obvious cytotoxicity in 40 µg/ml PCC NPs without irradiation. However, once irradiation was applied, cell viability showed a significant negative correlation with the concentration of PCC NPs. A colony formation assay further demonstrated the *in vitro* antitumor effect in three gastric cancer cell lines. The colony number in the treatment of PCC NPs was significantly less than that in photothermal treatment or chemotherapy (Figures 5C, D and Supplementary Figures S1 B, D). Flow cytometry was used to evaluate the effects of triggering apoptosis under different treatments. Figure 6E shows the flow cytometry result for the treatments. The PCC NPs treated group exhibited more significant apoptosis in SGC-7901 cells than the other group. The results indicated that PCC NPs could efficiently exert photothermal-chemotherapeutic synergy in gastric cancer cells.

### *In Vivo* Toxicity Evaluation of PCC NPs

PCC NPs were administrated via intravenous injection. Initially, the impact of PCC NPs in red blood cells was evaluated. The results of the hemolysis assay are shown in Figure 6A. Triton X-100 as the positive control caused more than 70% of red blood cells to hemolyze, which indicated that severe plasmorrhexis occurred under the treatment. The lysis rates of DOX and PCC NPs were both lower than 10%. This result suggests that PCC NPs do not cause hemolysis. Lysis in the IR-820 treatment accounted for nearly 20% of the total. One possible reason of this result is that the solvent of IR-820 was DMSO, which caused a degree of lysis. Then, the acute toxicity of PCC NPs was evaluated in BALB/c mice. The survival rate is shown in Figure 6B. Although the dose of DOX was only 3 mg/kg, it still exhibited obvious toxicity. Mortality was 60%. Two mice died after IR-820 treatment on day 1, possibly due to the solvent. IR-820 was dispersed in a mixture of DMSO and saline. The injection dose of PCC NPs was 100 mg/kg, but mice in the group still showed the highest survival rate. Pathological sections are displayed in Figure 6C. The results further demonstrated the safety of PCC NPs. DOX could cause severe myocarditis. Pathological slides of the heart in the DOX treated group show obvious typical characteristics of myocarditis. In the area where the arrow points, myocardial tissues were filled with lymphocytes. Moreover, hepatic tissue was also damaged by DOX treatment. The PCC NPs treated group also exhibited slight lymphocyte infiltration in cardiac and hepatic tissues. *In vivo* toxicity demonstrated that PCC NPs possess excellent biocompatibility.

### *In Vivo* Distribution of PCC NPs

**FIGURE 7:**
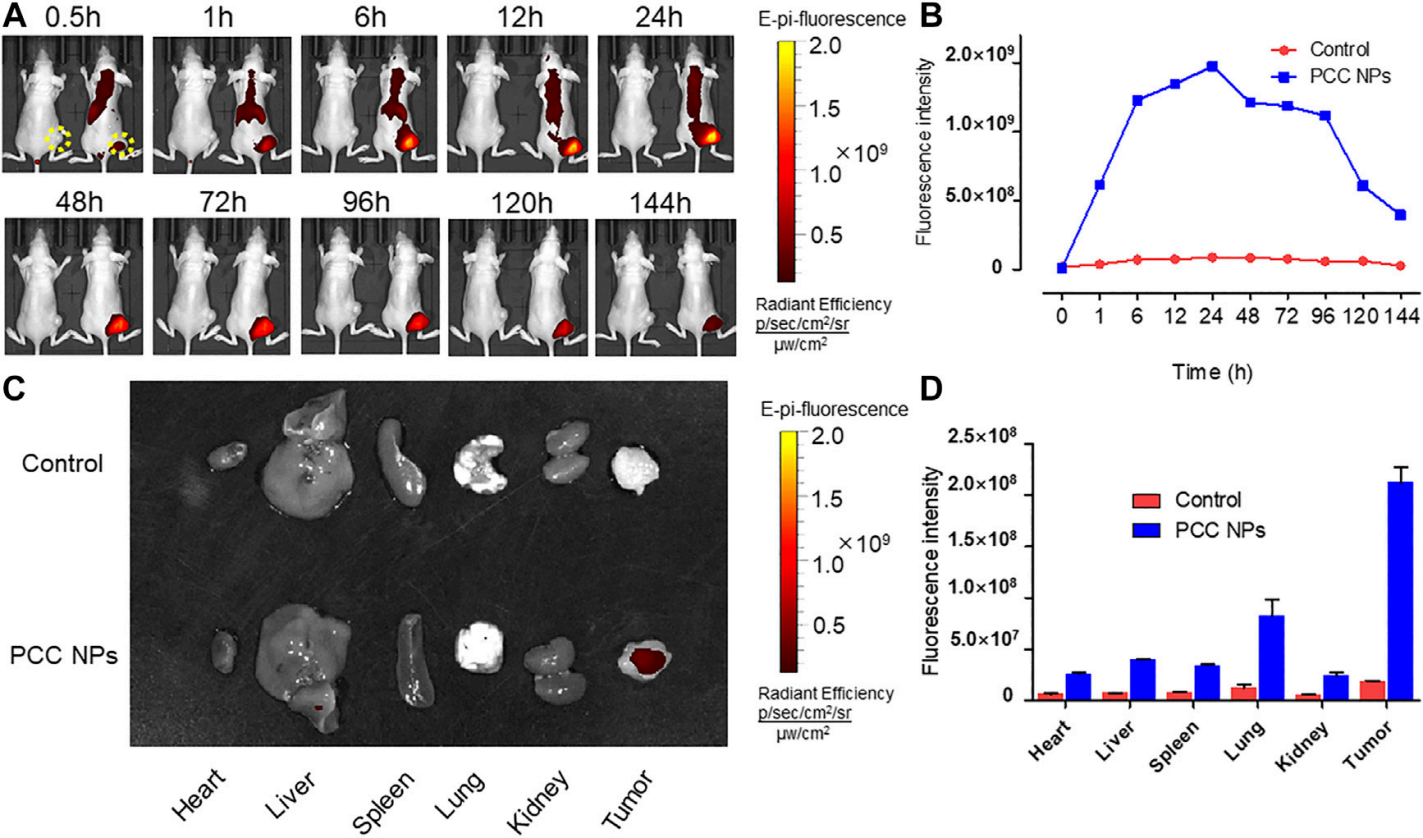
*In vivo* distribution and long-circulation of PCC NPs. The distribution of the NIR fluorescent signal in mouse **(A)**; the fluorescent intensity in tumor at different time **(B)**; the distribution of NIR fluorescent signal in organs and tumor tissues **(C)**; the fluorescent intensity in organs and tumor tissues **(D)**. Error bars represent the SD of the mean.

The *in vivo* distribution of PCC NPs was investigated using the Caliper IVIS Lumina II system (PE, USA). The results are shown in Figure 7. The continuous observation is exhibited in Figure 7A. The tumors of the control mouse were filled with a low fluorescent signal. In PCC NPs treated mouse, fluorescent signal initially appeared in the thorax and epigastrium and was then observed in the tumor area. Subsequently, the fluorescence continuously increased in the lung, liver, and tumor areas. The cumulative peak of the fluorescent signal in the tumor area appeared at approximately 24 h. Subsequently, fluorescence has declined across the whole body. Remarkably, the fluorescent signal remaining in tumor tissue was much more stable than that in other areas. The signal was continuously monitored in the tumor until 144 h, which means that PCC NPs could extend the circulation time of the payloads. The organs and tumor tissues were collected and observed. All tissues of control mouse had very low fluorescent signals, while the signals in PCC NPs treated mouse were still dense, especially in tumor. The results are shown in Figures 7C,D. The results amply demonstrate that PCC NPs could effectively increase accumulation of payload in tumor.

### *In Vivo* Antitumor Evaluation of PCC NPs

**FIGURE 8:**
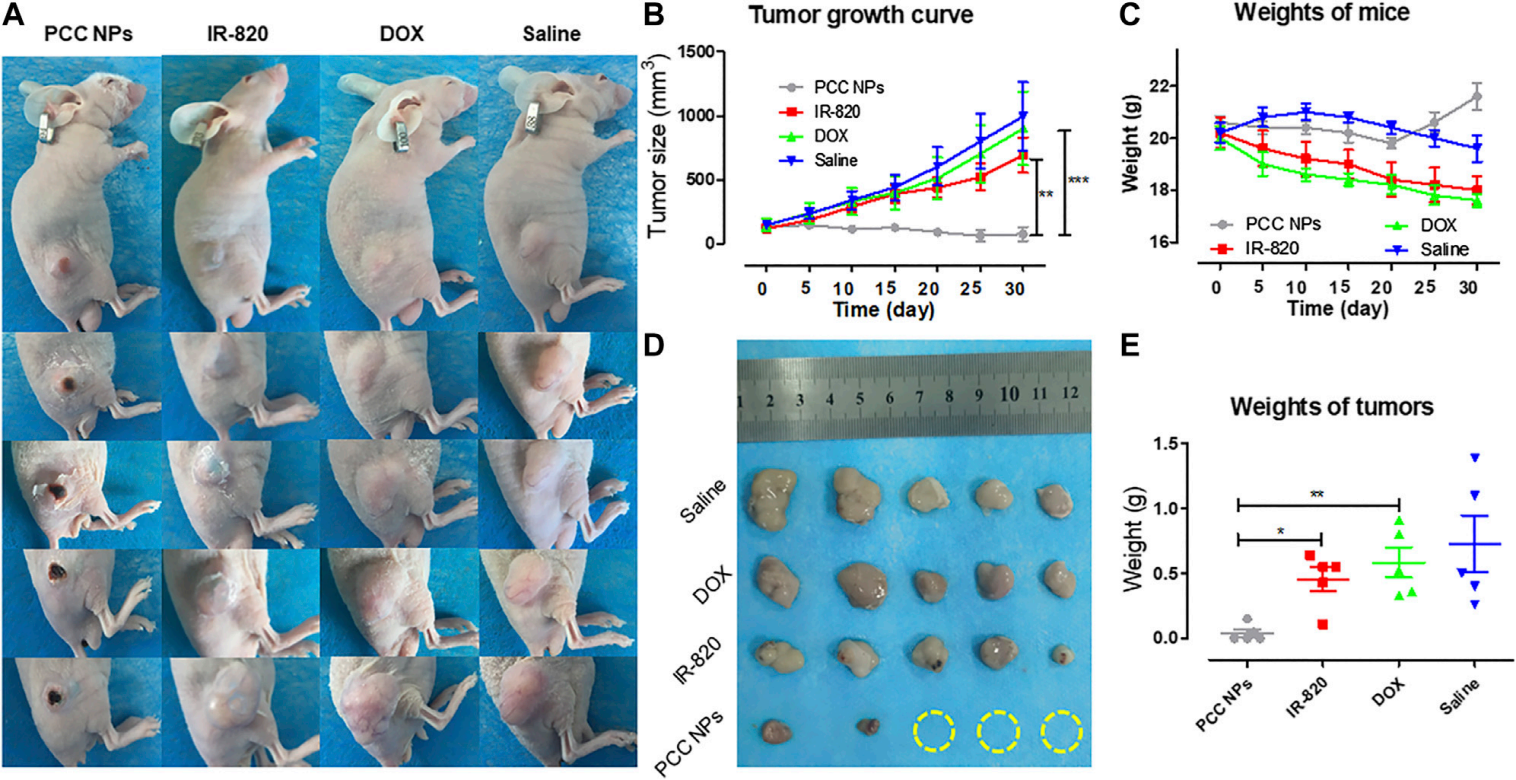
*In vivo* antitumor evaluation of PCC NPs. Mice photos under continuous monitoring **(A)**; the average volume of tumor in different treatment groups during the experiment **(B)**; the average weight of mice in different treatment groups during the experiment **(C)**; the tumor tissues from each treated group **(D)**; the average weight of tumor tissues from each treated group **(E)**. Error bars represent the SD of the mean. * indicates *p* < 0.05, ** indicates *p* < 0.01, and *** indicates *p* < 0.001.

To demonstrate the synergistic antitumor effect of PCC NPs, we randomly divided twenty gastric cancer xenograft models into four groups, which included the PCC NPs treated group, chemotherapeutic group, photothermal treatment group, and untreated group. The visual effects and tumor growth curves of the treatments are shown in Figures 8A, B. The untreated group was injected with saline, and the tumor rapidly grew to a large volume within a month. The average size of tumors increased almost 9-fold. The tumors in the chemotherapeutic group also grew quickly. The suppression effect was hardly discernible. The primary reason was that dose of the DOX in the treatment was low and could not inhibit proliferation. The single photothermal treatment was also ineffective. Although tumor under the skin area exhibited slight injury, the average tumor volume was increased nearly 6-fold. It is worth noting that the PCC NPs treatment greatly suppressed the growth of gastric tumors. As shown in Figure 8A, the tumor in the PCC NPs treated group exhibited slight burn after the first treatment, and the burn wound gradually increased until the tumor area was completely necrotic. The sizes of the tumors in the PCC NPs group were decreased under treatment and three tumors were eliminated. The suppression effect of PCC NPs was significantly better than that of the DOX or IR-820 treatment (Figures 8B,D). The weights of tumors exhibited identical results. The results indicated that PCC NPs could effectively treat gastric cancer *in vivo*.

Moreover, the weights of mice reflect the advantage of PCC NPs. The average weight of mice in the PCC NPs treated group underwent a rebound in the fourth week. By comparison, the mice in the DOX treated and untreated groups suffered constant emaciation. The average weight of mice in the IR-820 treated group increased in the first ten days and then gradually decreased until the experiment was completed. The results further demonstrated that PCC NPs possess superior *in vivo* biosecurity.

## Discussion

Early gastric cancer clinically has no obvious characteristic manifestations and diagnosis is difficult; the 5-year survival rate of patients with advanced gastric cancer is lower than 20% (Song et al., 2017). Gastrectomy is a primary mode of therapy for gastric cancer. However, postoperative recurrence is the most common form of problem and the most important cause of death in advanced gastric cancer after radical dissection. Adjuvant chemotherapy is a major part of the comprehensive treatment for gastric cancer; however, insufficient effect still impedes improvements in prognosis (Lazar et al., 2018). Therefore, an effective treatment combined with an accurate assessment of tumor progression is the most beneficial method in continuous therapy of gastric cancer. The combination of chemotherapy, photothermal therapy, and real-time monitoring can enhance curative effects in tumor treatment (Liu et al., 2011; Zheng et al., 2013; Chen et al., 2016; Li et al., 2016). For this purpose, a multifunctional nanoparticle, consisting of DOX and IR-820, was prepared via π-π stacking of PDA. PCC NPs have a distinctly spherical morphology and could be monodispersed under various aqueous conditions, such as PBS, FBS, and medium. The hydrodynamic diameter was 59.4 ± 3.6 nm, which allows tumor targeting via the EPR effect (Iyer et al., 2006). PCC NPs possess a superior encapsulation ability, which increased the bioavailability of the agents. The maximum ER of DOX and IR-820 were both over 96%, and the total DL was more than 37%. PCC NPs efficiently dispersed under physiological conditions and exhibited extraordinary stability. The release profile is another advantage of PCC NPs. There is a problem in pharmaceutical research, which is the trouble of the initial burst release of drugs. π-π stacking can realize sustained and controlled release of a loaded agent while ensuring better encapsulation and effectively avoiding burst release. The payload in PCC NPs could be stably encapsulated under the neutral conditions. Once the NPs enter an acidic environment, such as tumor tissues or lysosomes, the drugs will be released gradually (Wang et al., 2018). Meanwhile, when photothermy triggered, the payloads are also released from the NPs. The photothermal effect is crucial component of PCC NPs that not only directly injure tumor, but can also improve chemotherapy to meet the synergistic treatment of gastric cancer. In this study, PCC NPs have been proven to have superior photothermal conversion under laser irradiation. The NPs exhibited a rapid heating effect under irradiation. The maximum temperature was approximately 58°C and exhibited an obvious concentration dependence. The photothermal effect of PCC NPs was not impacted by the dispersed conditions. Moreover, the maximum temperature under the skin of the mouse reached approximately 47°C which is well suited for *in vivo* tumor thermotherapy and does not injure normal tissues (Hildebrandt et al., 2002). These results amply verify that PCC NPs possess excellent synergistic effect, which are appropriate in the treatment of gastric cancer.

Toxicity is the overriding factor regarding whether PCC NPs can be used in the treatment of gastric cancer. A series of evaluations were employed to verify the safety of PCC NPs. Initially, the cytotoxicity of the NPs was evaluated in six cell lines. PCC NPs were given as an injection through a vein. Thus, HUVECs, human umbilical vein endothelial cells, were used to initially evaluate injury to blood vessels. HL-7702 and IMR-90 are human normal liver cells and human embryonic lung fibroblast cells, respectively. These cells were employed to preliminarily evaluate whether PCC NPs could injure the liver and lung, which are most common cumulative organs of NPs. Moreover, three gastric cancer cell lines (BGC-823, SGC-7901, and MKN45) were used to determine the essential cytotoxicity of PCC NPs without irradiation. The results indicated that IR-820 has low cytotoxicity and DOX treatments exhibited more cytotoxicity than the PCC NPs treated groups; however there was no significant difference between the two treatments. Sustained release of DOX caused slightly low cytotoxicity of PCC NPs. Thus, biocompatibility was preliminarily verified *in vitro*. Subsequently, *in vivo* toxicity was evaluated using a hemolysis assay and according to acute toxicity. The NPs enter the circulation system first; thus, a hemolysis assay was used to measure the impact of PCC NPs in red blood cells. The lysis rate in the PCC NPs treated group was lower than 10%, suggesting that the NPs do not cause severe hemolysis. After the acute toxicity test, only two mice died in the PCC NPs injected group after 2 weeks. By comparison, the dose of DOX was only 3 mg/kg, but mortality reached 60% in DOX treated mice. Pathological analysis of the main organs further verified the safety of PCC NPs. Moreover, continuous *in vivo* administration also proves that PCC NPs possess superior *in vivo* biosecurity. The weights of the mice increased after PCC NPs treatment for 3 weeks. In contrast to the PCC NPs treated group, the mice in the DOX, IR-820, and saline treated groups suffered varying degrees of emaciation. These results indicated that PCC NPs could decrease systemic toxicity *in vivo* and exhibited excellent biocompatibility.

Continuous monitoring of gastric cancer is one of the main functions of PCC NPs. *In vitro* and *in vivo* imaging experiments were used to demonstrate whether PCC NPs could be used as probes for postoperative monitoring of gastric cancer. The cell internalization process showed that the PCC NPs could stably deliver dyes into the cell and then undergo intracellular release. The *in vivo* monitoring effect of PCC NPs was investigated by live imaging technology. The time-intensity curve, peak time, and tissue accumulation of the mouse were analyzed. At 30 min, the fluorescent signal synchronously appeared in the thorax, epigastrium, and tumor area of PCC NPs treated mouse and then continuously increased in these areas. After 24 h, the fluorescent peak in the tumor area appeared. Subsequently, the fluorescence gradually decreases. It is worth noting that the fluorescent signal was continuously observed in tumor over 6 days, and by comparison, the fluorescent signal remaining in tumor tissue was more stable than that in thorax and epigastrium. Subsequently, the main organs and tumors were observed and measured. The fluorescent signal in PCC NPs treated mouse was still dense, especially in tumor tissue. The results mean that PCC NPs could extend the circulation time of the payloads to large degree. Wang et al. reported ultralong circulating lollipop-like NPs, which are constructed with polydopamine, DOX, and gossypol via π-π stacking. The fluorescent signal remained in tumor tissues for 8 days. And the *in vivo* pharmacokinetic parameters of agents were greatly enhanced by the NPs (Wang et al., 2018). Postoperative continuous monitoring of gastric cancer could be performed by PCC NPs via NIR fluorescent imaging. Therefore, PCC NPs could be used to probe for accurate assessment of tumor progression. The ability will provide reliable information on the dynamic process of treatment, which can be used to determine or modify the appropriate therapeutic schedule against gastric cancer.

Many researchers have focused on chemo-photothermal combinations in cancer therapy (Hauck et al., 2008; Zhang et al., 2014). The suppression of PCC NPs was finally evaluated in a gastric cancer cell line and xenograft mouse model. SGC-7901 cells exhibited obvious damage after treatment with PCC NPs. The cellular morphology rapidly changed under irradiation; the vast majority of cells were abnormal. By comparison, the cells did not exhibit significant morphological changes in the unirradiated area. Meanwhile, the results indicated that cell viability showed a significant negative correlation with the concentration of PCC NPs under irradiation. Both colony formation and flow cytometry assays demonstrated that treatment with PCC NPs was significantly stronger than single photothermal treatment or chemotherapy. The results indicated that PCC NPs could effectively exert synergistic effects of chemotherapy and photothermal therapy against gastric cancer cells. Subsequently, *in vivo* antitumor evaluation was further employed to determine the synergistic effect of PCC NPs. Satisfyingly, PCC NPs treatment greatly suppressed the growth of gastric cancer in xenograft models. After treatment with PCC NPs, the tumor exhibited a slight burn within a short time; and then the burn wound gradually increased to fill the entire tumor area; finally, the tumor tissues became completely necrotic. Following continuous treatment with PCC NPs, the average volume of the tumors gradually decreased, and three tumors eliminated. The *in vivo* suppression effect of PCC NPs was also significantly better than that of single chemotherapy or photothermal treatment. The results amply demonstrated that PCC NPs could effectively treat gastric cancer.

## Conclusion

In conclusion, multifunctional NPs consisted of dopamine, poloxamer, DOX, and IR-820 via π-π stacking for the synergistic treatment of gastric cancer. PCC NPs possess a spherical morphology and good monodispersity. Meanwhile, the NPs exhibit a superior encapsulation ability, extraordinary stability, and pH-response release. *In vitro* photothermal conversion indicated that PCC NPs could effectively heat up under irradiation, and the temperature was suited to photothermal therapy. The biosecurity of the NPs was verified on six cell lines and BALB/c mice. The *in vivo* imaging results demonstrate that PCC NPs can perform continuous monitoring of gastric cancer. The fluorescent signal in tumor tissues was maintained for nearly 1 week after one injection. *In vitro* and *in vivo* antitumor experiments finally verified that PCC NPs possess an effective synergistic effect against gastric cancer. The present study can provide a theoretical basis for the development of a novel postoperative treatment method for gastric cancer.

## Data Availability Statement

The raw data supporting the conclusions of this article will be made available by the authors, without undue reservation.

## Ethics Statement

The animal study was reviewed and approved by the Laboratory Animal Administration Committee in Xi’an Medical University.

## Author Contributions

YZ and XZ designed the study. YZ and XS performed the experiments. YZ, XS, LZ, and XZ analyzed the results and data. YZ and XS prepared the manuscript. XZ and LZ modified the manuscript.

## Funding

This study was supported, in part, by the Youth Program of National Natural Science Foundation of China (81801863) and Innovation Capability Support Program of the Shaanxi Province (2020KJXX-050).

## Conflicts of Interest

The authors declare that the research was conducted in the absence of any commercial or financial relationships that could be construed as a potential conflict of interest.
