# Supplementary material for: pH-Sensitive and Long-Circulation Nanoparticles for Near-Infrared Fluorescence Imaging-Monitored and Chemo-Photothermal Synergistic Treatment Against Gastric Cancer
Source: Front Pharmacol. 2020 Nov 26;11:610883. doi: 10.3389/fphar.2020.610883 (PMC7768901; doi:10.3389/fphar.2020.610883)
Supplement: Supplementary file 1 [file image1.pdf]

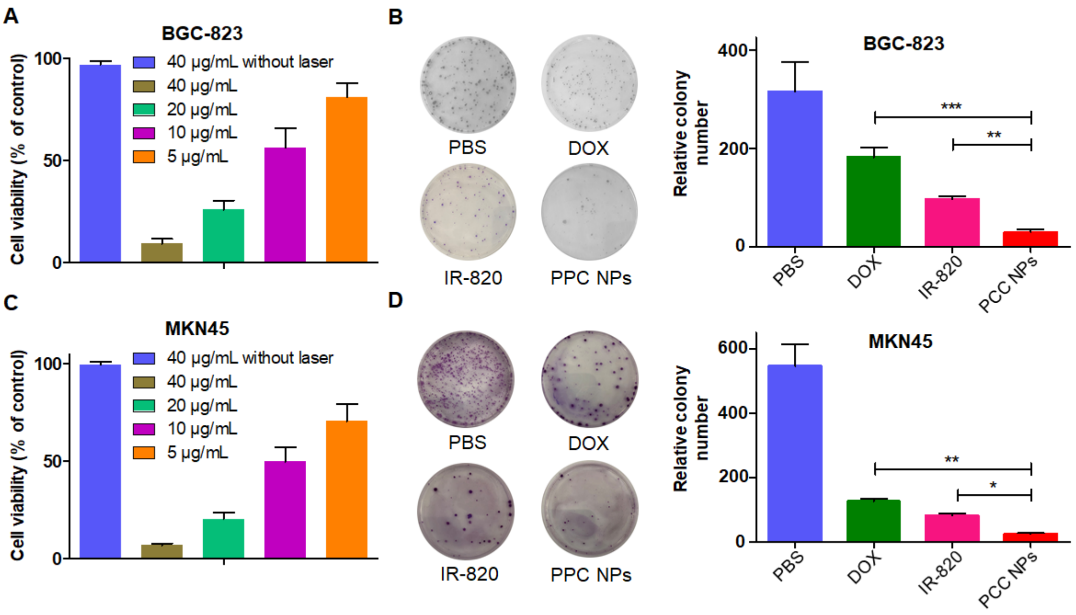


Figure S1. *In vitro* antitumor effect of PCC NPs in BGC-823 and MKN45 cells. The viability of BGC-823 cell at different concentrations of PCC NPs under photothermal treatment (A); the results of colony formation assays. BGC-823 cells in the PCC NPs and IR-820 treated groups were irradiated after the administration of samples (B); the viability of MKN45 cell at different concentrations of PCC NPs under photothermal treatment (C); the results of colony formation assays. MKN45 cells in the PCC NPs and IR-820 treated groups were irradiated after the administration of samples (D). The * indicates $p < 0.05$, ** indicates $p < 0.01$, *** indicates $p < 0.001$.
